# Clinical Characteristics and Risk Factors of *Corynebacterium striatum* Infection in Normally Sterile Body Fluids and Urine Specimens

**DOI:** 10.1155/cjid/9916536

**Published:** 2025-12-28

**Authors:** Yao Jiang, Yan Lei, Jing Liu, Xiaolan Guo, Hong Zhang

**Affiliations:** ^1^ Department of Clinical Laboratory, Affiliated Hospital of North Sichuan Medical College, Nanchong, China, hospital-nsmc.com.cn; ^2^ School of Laboratory Medicine, North Sichuan Medical College, Nanchong, China, nsmc.edu.cn; ^3^ Translational Medicine Research Center, North Sichuan Medical College, Nanchong, China, nsmc.edu.cn

**Keywords:** clinical characteristics, *Corynebacterium striatum*, risk factors, sterile body fluid, urine

## Abstract

**Background:**

*Corynebacterium striatum* (*C. striatum*) is an emerging opportunistic pathogen. Its role in pathogenicity can be difficult to determine and requires careful analysis of laboratory results and clinical symptoms. This research aims to investigate the clinical characteristics and risk factors of *C. striatum* infection in normally sterile body fluids and urine.

**Methods:**

A retrospective study was conducted in the Affiliated Hospital of North Sichuan Medical College in China. The study included 50 patients infected with *C. striatum* and 70 cases with non‐*C. striatum* infections from January 2018 to December 2023. Clinical data were analyzed, and independent risk factors for *C. striatum* infection were identified using chi‐square tests and multivariate logistic regression.

**Results:**

Patients in the *C. striatum* group were primarily distributed in the intensive care unit (ICU), neurosurgery, and nephrology departments. Most had a history of severe underlying diseases (80%), invasive procedures (84%), and antibiotic use (74%). The highest detection rate of *C. striatum* was 0.45% in 2018. Compared with the non‐*C. striatum* infection group, the *C. striatum* infection group showed a longer duration of invasive procedures, more days of antibiotic use, and a higher number of antibiotics used (*p* < 0.05). Logistic regression analysis identified them as independent risk factors for *C. striatum* infection in normally sterile body fluids and urine.

**Conclusion:**

Patients with severe conditions requiring multiple invasive procedures and prolonged treatment with multiple antibiotics may be more susceptible to *C. striatum* infection in normally sterile body fluids and urine. Clinicians should be aware of these risk factors to help prevent and manage *C. striatum* infections more effectively.

## 1. Introduction

*Corynebacterium striatum (C. striatum)*is an aerobic, nonspore forming, Gram‐positive bacterium that primarily colonizes human skin and the nasopharynx. Historically considered as a contaminant when detected in laboratory samples, its pathogenicity has gained recognition in recent years due to increased isolation from clinical specimens. Lee et al. found that *C. striatum* accounted for 13.3% of severe hospital‐acquired pneumonia cases [1]. In addition, metagenomic next‐generation sequencing (mNGS) identified *C. striatum* as the dominant bacterium in intensive care unit (ICU) patients with pulmonary infections, associated with poor prognosis [2]. Beyond common lower respiratory tract infections, *C. striatum* has been implicated in various sterile site infections, including meningitis, infective endocarditis, and bloodstream infection in patients with end‐stage renal diseases [3–5]. The 90‐day mortality rate for *C. striatum* patients with hematologic malignancies was reported to be 34% [6], underscoring its potential to cause adverse medical outcomes. However, most studies have been limited to case reports, so large‐sample, multisite research is needed to further elucidate the pathogenic ability of this bacterium [7, 8].

*C. striatum* primarily causes nosocomial infections and has been linked to severe clinical outbreaks [9, 10]. The lack of unified guidelines, coupled with its multidrug resistance, presents significant clinical challenges [11, 12]. Genomic analysis has revealed multiple drug‐resistant genes such as t*etW*, *ermX*, and *sul1*, but the mechanism of its drug resistance is still under investigation [13, 14]. While vancomycin and linezolid are commonly used treatments, multidrug resistance often leads to treatment failure [15], emphasizing the importance of early diagnosis.

Accurately determining the pathogenicity of *C. striatum* remains a critical issue for laboratory personnel and clinicians. Misidentification as a pathogen can lead to antibiotic abuse and drug resistance, while failing to recognize true infections can delay crucial treatment. Infections in sterile sites such as blood and the abdominal cavity often result in poor patient outcomes [16, 17]. Therefore, the focus should be on accurately diagnosing *C. striatum* infections in sterile body liquid. Accordingly, this study aims to analyze the clinical characteristics and risk factors of *C. striatum* infection in normally sterile body fluids and urine, comprehensively assessing its pathogenicity through both clinical and laboratory results. The findings can provide a theoretical basis for early prevention, control of nosocomial transmission, and improved identification of *C. striatum* infections.

## 2. Methods

### 2.1. Study Design

This retrospective cohort study was conducted from January 2018 to December 2023 at the Affiliated Hospital of North Sichuan Medical College, a 2500‐bed tertiary teaching hospital in China. The subjects were inpatients whose clinical isolates from normally sterile body fluids and urine specimens tested positive for *C. striatum* or other non‐*C. striatum*‐confirmed hospital‐acquired infections. Duplicate strains from the same patient and site were eliminated. Patient data were retrieved from the medical record database. The study was approved by the Ethical Committee of the Affiliated Hospital of North Sichuan Medical College in 2023 (2023ER456‐1), with the requirement for patient informed consent waived due to its retrospective nature.

### 2.2. Clinical Data

Clinical patient data were extracted from the medical record database, including (1) basic information: gender, age, body temperature, length of hospital stay, and admitting department; (2) clinical diagnosis; (3) preinfection medication use, including glucocorticoids, immunosuppressants, antibiotics, and duration; (4) invasive operations prior to infection, such as catheterization, serous cavity drainage, invasive ventilation, and surgery; (5) preinfection ICU stay duration; (6) postinfection medicine treatment; (7) clinical outcome during hospitalization refer to the patients’ status at discharge: a good outcome is defined as achieving the expected or ideal clinical state after treatment, including survival and disease remission, and a poor outcome is defined as failure to meet treatment goals or the occurrence of adverse clinical events, including death or treatment failure; and (8) laboratory examinations: procalcitonin (PCT), C‐reactive protein (CRP), white blood cell count (WBC), etc.

### 2.3. Definition

The cases in the study included 120 blood, cerebrospinal fluid (CSF), abdominal effusion (AE), pleural effusion (PE), and urine samples. Fifty *C. striatum*‐infected samples were designated as the *C. striatum* group. The remaining 70 non‐*C. striatum* cases, matched by the specimen type, department, and period, formed the non‐*C. striatum* group. The enrollment criteria for this study were as follows: (1) infection‐related indices: PCT ≥ 0.5 ng/mL, CRP ≥ 10 mg/L, WBC > 10 ∗ 10^9^/L or < 4 ∗ 10^9^/L, or increased leukocyte esterase or WBC in urine [18], and polymorphonuclear cells predominated in the CSF, AE, and PE, or decreased glucose concentration; (2) clinical symptoms: fever, chest or abdominal pain, high cranial pressure, or frequent, urgent, and painful urination, or renal pain [19–22]; (3) all isolated pathogens in these patients were confirmed as hospital‐acquired infections, with definitive diagnoses established at discharge; and (4) bacterial cultures from all 50 patients in the experimental group yielded pure growth of *C. striatum*. The exclusion criteria were as follows: (1) the isolates were considered contaminating or colonizing bacteria; (2) preadmission pregnancy or infection diagnosis; (3) incomplete or missing patient data; and (4) age < 18 years.

### 2.4. Bacterial Identification

Blood, CSF, AE, and PE samples’ collection adhered to surgical operation guidelines, with transport and culture methods following the Technical Requirements for Clinical Body Fluid Examination from the hygienic standard of the People’s Republic of China [23]. Isolated strains were cultured on blood agar plates (Pangtong, Chongqing, China) for 18 h at 37°C and identified by using MALDI‐TOF mass spectrometer (bioMérieux, France) with 99% identity.

### 2.5. Statistical Analysis

SPSS Statistics, Version 22.0 (IBM Corporation, Armonk, NY, USA), was used for data analyses. In the univariate analysis, continuous data were analyzed using a *t*‐test, while the categorical data were compared using a chi‐square test. *p* < 0.05 was considered statistically significant. Binary logistic regression analysis was used for multivariate analysis, with *p* < 0.05 indicating a significant risk factor associated with *C. striatum* infection.

## 3. Results

### 3.1. Patients and Clinical Characteristics

The study included 50 patients with *C. striatum* cultured from normally sterile body fluids and urine: 24 males (48%) and 26 females (52%), with an average age of 64 years old (range: 20–91 years). Patients were distributed across various departments, with the highest numbers in the ICU, neurosurgery, and nephrology. The most common underlying diseases were cerebrovascular disease, respiratory infection, and renal failure. Most patients (74%) had used antibiotics before infection, with 67.57% having used two or more antibiotics. Most patients (84%) had undergone more than one invasive procedure before infection (Table 1). The mortality rate in the *C. striatum* group was 8%, significantly higher than the rate of 4.29% (3/70) observed in the non‐*C. striatum* group.

**Table 1:** Clinical characteristics of patients with *C. striatum* in normally sterile body fluids and urine samples in the past 6 years.

Characteristics	Number of cases	Detection rate (%)
Gender		
Male	24	48
Female	26	52
Age (year)		
> 60	32	64
< 60	18	36
Distributed department		
ICU	7	14
Neurosurgery	6	12
Nephrology	6	12
Others	31	62
Underlying disease		
Cerebrovascular disease	11	22
Respiratory infection	10	20
Renal failure	7	14
Trauma	5	10
Kidney stones	5	10
Malignant tumor	4	8
Chronic liver disease	3	6
Cardiovascular disease	2	4
Intestinal perforation	2	4
Diabetes mellitus	1	2
Medication use		
Hormone use before infection	9	18
Antibiotics use before infection	37	74
Invasive manipulation		
Indwelling catheter	26	52
Serous cavity drainage	19	38
Surgery	14	28
Invasive ventilation	12	24
Others	3	6
Outcome at discharge		
Good clinical outcome	32	64
Poor clinical outcome	14	28
Death	4	8

### 3.2. The Positive Detection Rate of *C. striatum*

The annual positive detection rates for *C. striatum* across five types of specimens are shown in Table 2. From 2018 to 2023, the highest detection rate was in 2018 at 0.45%, while there were only 2 *C. striatum* cases in 2019. *C. striatum* was detected in 2 out of 17 PE specimens in 2022, the highest rate for a single specimen type. The third quarter consistently showed the lowest number of positive specimens, accounting for 12% of the total. Over the 6‐year period, the overall proportion of patients with *C. striatum* infection was 0.41% (Table 2).

**Table 2:** The positive detection rate of *C. striatum* in the past 6 years.

Specimen type	Year	Total number of cases	Number of cases				Detection rates (%)
			First quarter	Second quarter	Third quarter	Fourth quarter	
Blood	2018	426	1	0	0	1	0.46
	2019	468	1	0	0	1	0.03
	2020	290	1	1	0	0	0.69
	2021	528	0	1	0	0	0.19
	2022	560	1	0	1	0	0.36
	2023	641	0	3	1	0	0.62

Cerebrospinal fluid	2018	38	3	0	0	1	10.52
	2019	27	0	0	0	0	0
	2020	23	0	1	0	0	4.34
	2021	34	0	1	0	0	2.94
	2022	25	0	0	0	0	0
	2023	26	0	0	0	0	0

Pleural effusion	2018	20	0	0	0	1	5
	2019	20	0	0	0	0	0
	2020	18	0	0	0	0	0
	2021	29	0	0	0	2	6.89
	2022	17	1	0	1	0	11.76
	2023	33	0	0	0	0	0

Abdominal effusion	2018	23	0	0	0	0	0
	2019	63	0	0	0	0	0
	2020	21	0	0	0	0	0
	2021	166	0	1	0	0	0.60
	2022	162	0	0	1	0	0.62
	2023	213	1	0	0	0	0.46

Urine	2018	1052	0	0	0	0	0
	2019	1478	0	0	0	0	0
	2020	1312	0	0	0	0	0
	2021	1740	3	1	0	1	0.29
	2022	1425	2	2	2	2	0.56
	2023	1400	3	7	0	0	0.71

Total		12,278	17	18	6	9	0.41

### 3.3. *C. striatum* Infection Based on Chi‐Square Tests

A control group of patients with non‐*C. striatum* was selected, matched for the specimen type, department, and period. Clinical information of all enrolled patients is provided in Supporting Table (available here). There were no differences in age, sex, and length of hospital stay between the two groups. However, the duration from the invasive operation to the detection of pathogenic bacteria in the *C. striatum* infection group was longer than that in the control group. Furthermore, the *C. striatum* group had higher proportions of patients with ≥ 2 invasive operations, longer antibiotic use, and ≥ 2 antibiotic types used, with all differences statistically significant (*p* < 0.05). Laboratory parameters showed no significant difference between groups (Table 3).

**Table 3:** Comparison of risk factors in *C. striatum* and non‐*C. striatum* groups.

Variates	*C. striatum* group (*n* = 50)	Non‐*C. striatum* group (*n* = 70)	Statistical magnitude	*p* value
Age (year)	64.060 ± 2.036	63.843 ± 1.580	0.825	0.932
Sex (female)	26 (52%)	29 (41.43%)	1.313	0.270
Length of hospital stay (day)	30.85 ± 4.649	29.55 ± 2.917	0.547	0.696
Duration after invasive operation	21.59 ± 6.539	7.785 ± 1.256	4.487	0.009^∗^
Duration after invasive operation ≥ 7 (days)	33 (66%)	20 (28.57%)	16.569	0.001^∗^
Number of invasive operations ≥ 2 (times)	36 (72%)	37 (52.86%)	4.486	0.039^∗^
Days of antibiotic use	9.022 ± 1.245	3.862 ± 1.325	2.342	0.0218^∗^
Types of antibiotics used ≥ 2 (types)	31 (62%)	22 (31.43%)	11.054	0.001^∗^
PCT (ng/mL)	2.637 ± 1.323	3.081 ± 2.178	4.315	0.243
WBC (10^9^/L)	10.45 ± 0.8308	8.969 ± 0.827	0.040	0.312
*N* (10^9^/L)	8.481 ± 0.8407	7.429 ± 0.794	1.312	0.472
*N* (%)	75.620 ± 2.751	88.494 ± 12.093	0.531	0.325
RBC (10^12^/L)	3.350 ± 0.097	3.309 ± 0.092	0.148	0.809
HGB (g/L)	98.100 ± 2.740	93.984 ± 3.504	1.086	0.723
PLT (10^9^/L)	200.800 ± 17.820	182.619 ± 17.092	0.089	0.596
TP (g/L)	62.13 ± 1.899	62.406 ± 2.101	2.132	0.330
Alb (g/L)	33.12 ± 0.965	35.790 ± 2.239	2.704	0.407

*Note:* PCT, procalcitonin; N, neutrophil count; N%, neutrophil percentage; HB, hemoglobin; PLT, platelet; Alb, albumin.

Abbreviations: RBC, red blood cell; TP, total protein; WBC, white blood cell.

^∗^Significance level of *p* < 0.05 indicates statistical significance.

### 3.4. Infection of *C. striatum* Based on Multivariate Regression Analysis

**Figure 1:**
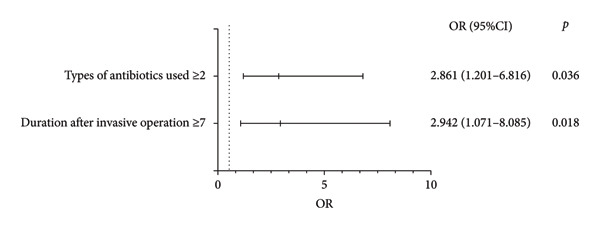
Multiple logistic analysis of risk factors of *C. striatum* in patients with infection of normally sterile body fluid and urine.

The categories showing significance in the univariate analysis were subjected to binary logistic regression. The results indicated that the duration after invasive operation ≥ 7 days and the use of antibiotics ≥ 2 types were independent risk factors for *C. striatum* infection in normally sterile body fluids and urine (Figure 1).

## 4. Discussion

According to the China Antimicrobial Surveillance Network (CHINET), in 2023, Gram‐positive bacteria accounted for about 36.1% of infections and Gram‐negative bacteria for about 63.9%. The increasing widespread drug resistance has become a global public health problem. Infection is reported to be one of the leading causes of death worldwide, with the emergence of multidrug‐resistant bacteria exacerbating this situation [24, 25]. The abuse of antibiotics is widely recognized as the primary cause of bacterial resistance, making rational application of antibiotics after pathogen identification crucial in curbing the emergence of resistant bacteria [26]. *C. striatum*, a normal flora that lives on human skin, has long been dismissed as a contaminant when cultured. However, recent studies have found that it can cause severe infections at multiple sites, resulting in adverse medical outcomes [27, 28]. Some medical units lack advanced technologies like gene sequencing or MALDI‐TOFMS for *C. striatum* identification, leading to irrational antibiotic use and aggravating the generation of drug‐resistant bacteria [1]. While high‐resolution melt curve analysis (HRM) has been developed to quickly identify *C. striatum*, it is not yet widely used as a routine detection method [29]. Given the colonization properties of *C. striatum*, comprehensive analysis is still needed to determine its pathogenicity after identification. This study explored risk factors for *C. striatum* infection in normally sterile body fluids and urine samples to provide reliable evidence for clinically confirming *C. striatum* as a pathogenic bacterium and guiding rational antibiotic treatment.

Among patients with *C. striatum* infection in normally sterile body fluids and urine, the sex ratio (male:female) is about 1:1, mainly concentrated in elderly patients. Most patients have severe cerebrovascular, respiratory, and renal system diseases, with the highest distribution in the ICU, neurosurgery, and nephrology departments. This may be due to the critical condition and low immunity of patients in these three departments, aligning with previous research findings [30]. Additionally, patients with *C. striatum* infection often have a history of antibiotic use and invasive procedures, including indwelling catheters, serous cavity drainage, mechanical ventilation, and surgery, creating opportunities for *C. striatum* to invade the human body. This suggests that patients in the ICU, neurosurgery, and nephrology departments with a history of multiple antibiotic use and invasive procedures should be closely monitored for *C. striatum* infection to avoid adverse consequences [9].

To further confirm the risk factors for *C. striatum* infection in clinical normally sterile body fluids and urine, control patients were collected with other pathogen cultures from the same hospital period, department, and specimen type as the *C. striatum* infection group. This approach minimized the influence of patient factors, departmental environment, and clinical treatment on the study results. This study found that the duration of invasive procedures, number of invasive manipulations, and amount of antibiotics used were greater in the *C. striatum* infection group compared with controls, consistent with previous studies [9, 31]. In addition, duration after invasive operation ≥ 7 days and use of antibiotics ≥ 2 types were identified as independent risk factors for *C. striatum* infection in normally sterile body fluids and urine. Some studies have found that admission to the ICU, length of hospital stay, hypoproteinemia, and hormone use are independent risk factors for respiratory *C. striatum* infection [9, 32, 33], which differs from the results of this study. This discrepancy may be due to differences in infection sites studied and control group inclusion criteria. In previous studies, patients colonized by *C. striatum* were included as the control group, which may come from different departments and have different disease backgrounds from the experimental group, likely causing inconsistent results. The results of this study suggest that *C. striatum* infection may be more likely to occur in patients with prolonged invasive procedures, multiple invasive procedures, and extensive antibiotic use. Clinicians should pay special attention to this susceptible group. This may be because *C. striatum*, a multidrug‐resistant bacterium, becomes the dominant bacterial group after antibiotic application. Its ability to adhere, invade, and form biofilms by binding fibrinogen to medical implants further promotes infection [34, 35]. Studies have shown that *C. striatum* from infection groups produces significantly more biofilm than colonization groups, attributed to pathogenic virulence factors such as neuraminidase, hyaluronidase, and hemolysin [36, 37]. These biofilms enhance antibiotic resistance, immune evasion, and survival in hospital settings, possibly due to the function of the extracellular matrix [38]. *C. striatum* readily develops resistance to disinfectants through biofilm formation. A study found that 2% peracetic acid and 1% potassium persulfate have better bactericidal effects than 2% glutaraldehyde although efficacy changed over time. Clinicians should understand disinfectant efficacy and select appropriate ones for medical devices and environments to effectively prevent hospital‐acquired infections [39].

Among the 50 normally sterile body fluids and urine samples collected in this study, although the proportion of positive cultures was not high, the detection rate of *C. striatum* has increased annually, peaking in spring and summer. PE samples had the highest detection rate, all associated with severe underlying diseases. Fourteen patients (28%) experienced poor clinical outcomes, and four (8%) died, a lower mortality rate than previously reported [12]. Most patients discharged with improvement were treated with vancomycin or linezolid, which is consistent with the findings of a review of 42 studies that recommended vancomycin as the first‐choice antibiotic, with linezolid or daptomycin for severe infections [12]. However, a study using Inverse Probability of Treatment Weighting (IPTW) found limited clinical efficacy and drug side effects for vancomycin and linezolid targeted therapy, suggesting the need for prospective studies [40]. Another study identified niclosamide as a potential therapeutic agent against *C. striatum* MDR infections [41]. These findings underscore the need for further exploration of new pharmacological treatments for *C. striatum* infection.

## 5. Conclusion

*C. striatum* isolates can cause severe infections in normally sterile body fluids and urine. Patients with severe underlying diseases and low immunity who undergo invasive operations and antibiotic treatment may be related with *C. striatum* infection. Our data provide new insight into evaluating the clinical significance of *C. striatum* cultures from normally sterile body fluids and urine. However, as a retrospective study, our findings can only demonstrate associations between exposure factors and clinical outcomes. More rigorous prospective studies or randomized controlled trials are warranted to validate these conclusions.

## Ethics Statement

All experimental protocols were approved by the Research Ethics Committee of the Affiliated Hospital of North Sichuan Medical College (2023ER456‐1). All methods were carried out in accordance with the Declaration of Helsinki.

## Disclosure

All authors approved the final version of the manuscript.

## Conflicts of Interest

The authors declare no conflicts of interest.

## Author Contributions

Yao Jiang, Hong Zhang and Xiaolan Guo designed and conceptualized the study. Yao Jiang and Jing Liu collected and analyze data. Hong Zhang performed statistical analysis. The manuscript was written by Yao Jiang and Yan Lei. Yao Jiang and Yan Lei contributed equally to this work.

## Funding

This research was supported by North Sichuan Medical College, CBY23‐QNA36.

## Supporting information


**Supporting Information** Additional supporting information can be found online in the Supporting Information section.

## Data Availability

The data that support the findings of this study are available from the corresponding author upon reasonable request.
